# Macrophage Infiltration, Activation, and Therapeutic Implication in Skeletal Muscle Injury and Repair

**DOI:** 10.3390/ijms27031332

**Published:** 2026-01-29

**Authors:** Xingyu Wang, Lan Zhou

**Affiliations:** Department of Neurology, Hospital for Special Surgery, 535 East 70th Street, New York, NY 10021, USA; zhoula@hss.edu

**Keywords:** monocytes, macrophages, inflammation, infiltration, muscle injury, muscular dystrophy

## Abstract

Skeletal muscle injury triggers inflammatory response, of which the accumulation of intramuscular monocytes/macrophages is a prominent feature. Macrophages in injured muscle comprise both blood monocytes-derived infiltrating macrophages, which are recruited through CCR2 signaling, and pre-existing muscle resident macrophages, which are established during embryogenesis and maintained until adulthood through self-renewal proliferation. During regenerative acute muscle injury, infiltrating monocytes/macrophages are heterogeneously activated in a temporal dynamic, responding to the changing microenvironment in injured muscle and contributing to the complete injury repair. Injury-associated monocytes/macrophages recede with the completion of muscle injury repair. In contrast, injury-associated monocytes/macrophages persist in dystrophic muscle of Duchenne muscular dystrophy (DMD), likely accounting for persistent inflammation and progressive fibrosis of DMD muscle. We review here the current knowledge on monocyte/macrophage infiltration and activation in both acutely injured skeletal muscle and dystrophic muscle with subsequent discussion of the potential therapeutic implication in treating muscular dystrophy.

## 1. Introduction

The homeostasis of skeletal muscle can be compromised by various factors, such as acute injury, infection, genetic defects, and aging. The consequences differ based on the triggering factors. Acute injuries from trauma or myotoxin exposure are usually repairable. However, triggering factors, such as repeated injuries, large volumetric muscle loss, and chronic muscle injury caused by genetic defects, commonly result in fibrofatty tissue deposition, impairing muscle function. Successful skeletal muscle regeneration hinges on a highly coordinated process involving myofiber regeneration, inflammation, extracellular matrix (ECM) remodeling, and revascularization [1,2,3]. Myofiber regeneration depends on muscle resident myogenic stem cells, muscle satellite cells (MuSCs) [4,5], which are activated and differentiated in response to injury-induced changes in tissue microenvironment to rebuild myofibers [6,7,8,9]. Among all the environmental factors impacting myogenesis, macrophages have emerged to be crucial in regulating the quiescence, activation, and differentiation of MuSCs in a spatiotemporal manner [10,11].

Macrophages are multi-originated cells, exhibiting remarkable functional heterogeneity, including their active roles in maintaining tissue homeostasis and repairing injured muscle [12,13,14,15,16]. In the steady state, resident macrophages act as surveillance cells, maintaining tissue integrity. Following acute injury, macrophages are essential for regenerative repair. However, in chronic or degenerative conditions, macrophages can contribute to tissue pathology [15,16,17,18,19], making them a potential therapeutic target. Currently, the origins of intramuscular macrophages both at the steady and disease states, as well as the chemotactic mechanisms of macrophage infiltration in the disease state, have been comprehensively elucidated. The heterogenic activation of macrophages is well documented in both acutely and chronically injured skeletal muscles. We review here the current knowledge on macrophage infiltration and activation in both regenerative (acute) and degenerative (muscular dystrophy) skeletal muscle injury and discuss its potential therapeutic implications to treat muscular dystrophy.

## 2. The Origins of Intramuscular Macrophages: Resident and Infiltrating Macrophages

Tissue macrophages are categorized into two main types: resident macrophages and infiltrating inflammatory macrophages. Resident macrophages, which populate all adult tissues including skeletal muscle, are primarily established during embryonic development [20]. They originate from two embryonic sources: primitive yolk sac macrophages and, subsequently, fetal liver monocytes (fetal monocytes). Fetal monocytes eventually replace most yolk sac-derived macrophages in nearly all tissues, except for the brain [14,21,22,23,24,25,26,27,28]. These embryonic macrophages can maintain themselves into adulthood through self-renewal. In the adult tissues including skeletal muscle, resident macrophage populations are further contributed to by adult blood monocytes, which originate from bone marrow hematopoietic stem cells (HSCs) and circulate in the blood [20,29,30,31,32,33]. In contrast to the resident macrophages, infiltrating macrophages are derived exclusively from HSC-originated adult blood monocytes [34,35]. While resident macrophages can be found in both steady-state and injured tissues, infiltrating macrophages are differentiated from recruited blood monocytes in injured tissues.

**Figure 1:**
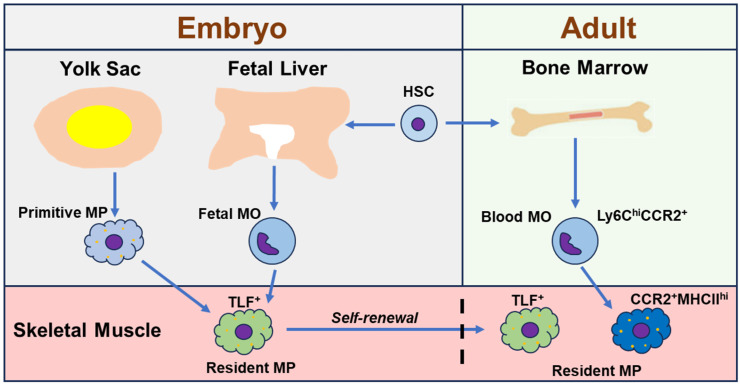
Scheme showing the origins of adult skeletal muscle resident macrophages. TLF^+^ muscle resident macrophages are established during embryo development from both yolk sac and fetal liver hematopoiesis. They persist into adulthood through self-renewal. CCR2^+^MHCII^hi^ muscle resident macrophages are from adult bone marrow HSC-derived blood Ly6C^hi^CCR2^+^ monocytes. HSC: hematopoietic stem cell. MO: monocyte. MP: macrophage. TLF^+^: Timd4^+^ and/or Lyve1^+^ and/or Folr2^+^.

Skeletal muscle resident macrophages have been identified and studied. In the steady-state skeletal muscle of adult mice, resident macrophages reside in interstitial tissue and can be identified as CD45^+^F4/80^+^CD64^+^ Ly6C^lo^ cells [20]. Lineage-tracing studies show that these muscle resident macrophages mostly originate from fetal monocytes and adult bone marrow HSCs, with only a small percentage from primitive macrophages [20]. The HSC-originated resident macrophages increase in fraction with aging, suggesting a gradual replacement of the embryo-originated resident macrophages by the HSC-originated ones [20]. Further studies with flow cytometry (FACS) and single cell-based RNA sequencing (scRNAseq) analysis reveal that embryo-originated resident macrophages are mainly of CCR2^-^MHCII^lo^Lyve1^hi^ and adult HSC-originated resident macrophages are completely of CCR2^+^MHCII^hi^Lyve1^lo^ [20]. These findings were confirmed by another study using parabiosis and single-cell transcriptome analysis, which identified similar muscle resident macrophage subtypes: self-renewal TIM4^+^Lyve1^+^CCR2^-^ cells and blood replenished TIM4^-^Lyve1^-^CCR2^+^ cells [36]. Furthermore, a recent study identified similar resident macrophage subpopulations across different steady-state murine tissues including skeletal muscle based on the expression of *Timd4*, *Lyve1*, *Folr2*, and *Ccr2* genes: (1) *Timd4*^+^ and/or *Lyve1*^+^ and/or *Folr2*^+^ (*TLF*^+^) self-renewal macrophages originated from yolk sac and fetal monocyte precursors; (2) *TLF*^-^*Ccr2*^+^ macrophages that can be entirely replaced by blood monocytes; and (3) *TLF*^-^*Ccr2*^-^ macrophages which express a high level of MHCII molecules and receive a moderate contribution from adult monocytes [37]. These findings together strongly suggest that *TLF* expression primarily marks the embryo-derived resident macrophages and CCR2 and MHCII expression preferentially mark the adult monocytes-derived resident macrophages at least in steady-state murine tissues. The origins of adult skeletal muscle resident macrophages are summarized in Figure 1. Interestingly, scRNAseq analysis of human macrophages also indicated that the *Lyve1*^+^ macrophages overlap strongly with fetal liver macrophages while the *Lyve1*^-^ macrophages expressed the monocyte signatures gene, suggesting embryonic and monocytic origins of *Lyve1*^+^ and *Lyve1*^-^ macrophages, respectively [38]. It therefore suggests that human tissue resident macrophages may share similar phenotypes with murine ones.

## 3. Macrophage Infiltration Following Skeletal Muscle Injury

### 3.1. Blood Monocytes Consist of Two Subtypes

Infiltrating macrophages are derived exclusively from HSC-originated adult blood monocytes following tissue injury [34,35]. Murine blood monocytes consist of two principal subsets: Ly6C^hi^CCR2^+^CX3CR1^lo^ inflammatory monocytes and Ly6C^lo^CCR2^-^CX3CR1^hi^ patrolling monocytes, distinguished by the expression of cell surface markers Ly6C, C-C motif chemokine receptor 2 (CCR2), and C-X3-C motif chemokine receptor 1 (CX3CR1) [39]. In humans, Ly6C is not expressed, and the CD14^hi^CD16^lo^ and CD14^lo^ CD16^hi^ monocytes correspond to the Ly6C^hi^ and Ly6C^lo^ monocytes in mice, respectively [40].

### 3.2. Infiltrating Macrophages Are Derived from Ly6C^hi^, but Not Ly6C^lo^, Blood Monocytes in Both Acutely Injured and Dystrophic Skeletal Muscles

Following acute skeletal muscle injury, Ly6C^hi^ inflammatory monocytes in blood circulation quickly migrate into injured muscles and differentiate into Ly6C^hi^ inflammatory macrophages [39]. The spleen serves as an additional reservoir for these Ly6C^hi^ monocytes, which can be mobilized to sites of inflammation including skeletal muscle [41,42]. This recruitment from the blood circulation depends on the chemokine system, where tissue cells release chemokine ligands that attract circulating cells bearing matching receptors. Specifically, the influx of Ly6C^hi^ monocytes into acutely injured muscle requires CCR2 on the monocytes and its primary ligand, CCL2, which is produced by both resident muscle cells and infiltrating macrophages [43,44,45]. Impairing CCR2 or CCL2 expression reduces macrophage accumulation in various acute muscle injury models [35,43,44,45,46]. In contrast, Ly6C^lo^ patrolling monocytes patrol the vascular endothelium and may enter tissues via CX3CR1/CX3CL1 signaling to contribute to resident macrophage populations [47]. But it has been shown that Ly6C^lo^ monocytes are not recruited by acutely injured muscle during injury repair [48]. Despite that, Ly6C^hi^ macrophages only dominate at day 1 post-injury, and Ly6C^lo^ macrophages become the predominant intramuscular macrophage type at day 3 post-injury, following acute muscle injury [35,44]. This shift occurs because Ly6C^hi^ inflammatory macrophages switch to a Ly6C^lo^ state after phagocytosing necrotic muscle debris [35,49,50,51,52,53]. This Ly6C^hi^-to-Ly6C^lo^ switch, rather than the pre-existing resident macrophages at steady state, is the major contribution to the Ly6C^lo^ macrophages accumulated in injured muscle [35,53,54].

As seen in acutely injured skeletal muscles, accumulation of macrophages is also a prominent feature in chronically injured muscles of Duchenne muscular dystrophy (DMD) mouse model *mdx* [55]. Blood Ly6C^hi^ monocytes are recruited into *mdx* muscles via CCR2 signaling [56,57]. Ly6C^lo^ monocytes do not contribute to intramuscular macrophages in *mdx* mice, even in the absence of CCR2 signaling [58]. Intramuscular Ly6C^lo^ macrophages in *mdx* muscles are also switched from Ly6C^hi^ macrophages [56,57,58]. Lineage-tracing and scRNAseq studies have provided evidence that macrophages in the dystrophic *mdx* muscles are mostly HSCs-originated and blood monocytes-derived infiltrating macrophages and that embryo-originated resident macrophages barely expand and contribute minimally to the intramuscular macrophages in *mdx* muscles [58].

## 4. Pro-Regenerative Macrophage Activation in Acute Skeletal Muscle Injury and Repair

Macrophage activation has been extensively studied in mouse models of acute skeletal muscle injury, which can be induced by multiple approaches, such as mechanical damage, contusion, freeze, myotoxin or heavy metal salt injection, or ischemia, and these injuries lead to a similar repair process [59]. Acute skeletal muscle injury usually repairs within 2 to 3 weeks without significant residual deficits unless the injury is repeated [60] or with large volume of muscle loss [61,62]. In the barium chloride (BaCl_2_)-induced limb muscle injury, massive myofiber necrosis along with infiltration of inflammatory cells is seen early after injury, such as on day 1 post-injury. Around day 3 post-injury, necrotic myofibers largely disappear, while inflammatory cell infiltration reaches peak. On day 7 post-injury, inflammatory cell infiltration decreases compared to on day 3. Meanwhile, necrotic myofibers are completely gone, central-nucleated regenerating myofibers are massively present, and deposition of extracellular matrix is seen. On day 14 post-injury, inflammatory cell infiltration completely recedes, regenerated myofibers grow in size, and deposition of extracellular matrix largely decreases [44]. An adequate inflammatory response, featuring massive macrophage infiltration, is required for the complete muscle regeneration [1,63,64,65]. Both muscle resident macrophages and infiltrating macrophages are required in this regenerative skeletal muscle injury repair [10,35,36,43,44,45,46,66,67].

### 4.1. Temporal Dynamics and Heterogeneity of Macrophage Subpopulations During Acute Skeletal Muscle Injury Repair

**Figure 2:**
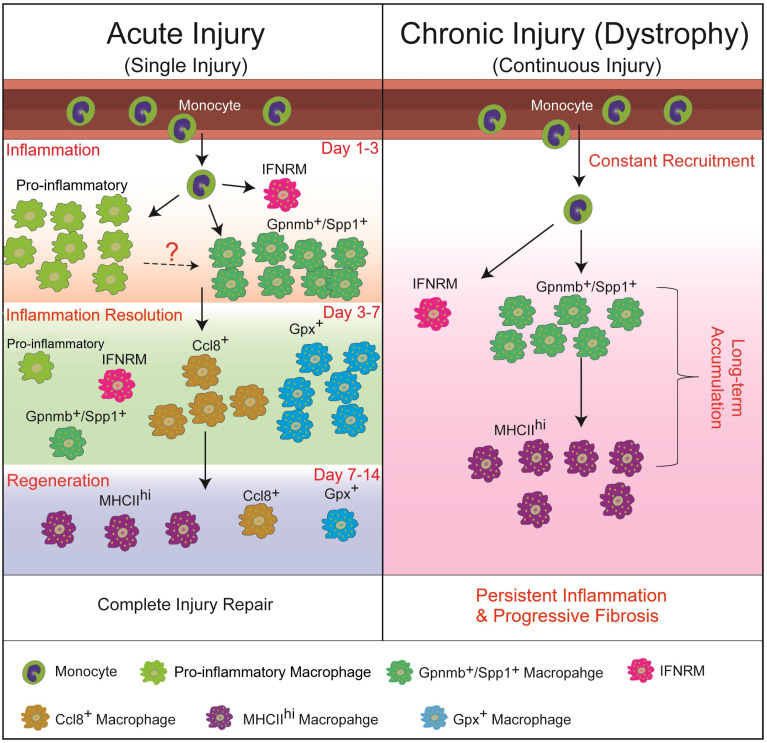
Schematic summary of the heterogeneous activation of infiltrating monocytes/macrophages in acutely injured and dystrophic skeletal muscle. Evidence is still lacking whether pro-inflammatory macrophages can further differentiate into Gpnmb^+^/Spp1^+^ macrophages or not. IFNRM: interferon-responsive macrophage.

The switch from Ly6C^hi^ to Ly6C^lo^ macrophages during skeletal muscle repair indicates significant shifts in activation status of these cells. Historically, Ly6C^hi^ and Ly6C^lo^ macrophage subpopulations were associated with the bipolar M1 (pro-inflammatory) and M2 (anti-inflammatory, pro-regenerative, or pro-fibrotic) classifications, respectively [68,69]. This phenotypic switch does coincide with a functional change from a pro-inflammatory to an anti-inflammatory state, as evidenced by the switches of pro- to anti-inflammatory gene expression [15,19,35,49,54,66,70,71,72] and pro- to anti-inflammatory lipid mediator production [73,74]. However, in vivo studies have strongly suggested that this M1/M2 dichotomy is over-simplified and insufficient to describe macrophage behavior in complex in vivo settings [68,75,76,77]. Macrophages are highly plastic and can rapidly adapt their function in response to the tissue microenvironment, resulting in diverse subtypes. Recent studies, particularly those with single cell-based transcriptome analysis, have revealed coexistence of heterogeneous macrophage subpopulations at various time points with dynamic change in proportions during acute skeletal muscle injury repair [78,79,80,81]. Early after injury (day 0.5 to 2), two monocyte/macrophage subpopulations predominate: one featuring the expression of genes resembling blood Ly6C^hi^ monocytes and the other one featuring pro-inflammatory genes (i.e., *Cxcl1, Cxcl3, Ccl2, Ccl6, Ccl7, Il1a, Il1b,* and *Cd14*). Around day 3 post-injury, monocytes are still present, while the pro-inflammatory subpopulation disappears, along with emergence of two macrophage subpopulations: (1) Gpmnb^+^Spp1^+^ subpopulation featuring genes regulating ECM remodeling (i.e., *Spp1, Fabp5,* and *Trem2*) and muscle regeneration (i.e., *Gpnmb, Igf1,* and *Gdf15*); (2) interferon-responsive macrophage (IFNRM) subpopulation featuring high expressions of IFN-responsive genes (ISGs) such as *Rsad2, Ifit1, Cxcl10,* and *Irf7*. Day 3 post-injury also sees massive proliferation of macrophages. After day 3, monocytes, Gpmnb^+^Spp1^+^, and proliferating subpopulations recede over time, along with emergence of macrophage subpopulations featuring inflammation-resolution genes (i.e., *Gpx3, Rgs2, Ccl8,* and *Mrc1*) and resident macrophage genes (i.e., MHCII genes) [78,81] (Figure 2). The temporal dynamics of different monocyte/macrophage subpopulations correlate with the different stages of acute skeletal muscle injury and repair: (1) inflammatory subpopulation emerges along with massive myofiber damage at day 1; (2) massive macrophage proliferation is seen at the peak of inflammation at day 3; (3) Gpmnb^+^Spp1^+^ and IFNRM subpopulations emerge after starting the clearance of necrotic myofibers at day 3 and precede deposition of ECM and emergence of regenerating myofibers; (4) inflammation resolution subpopulations emerge along with recession of inflammation from day 3 to day 7; (5) Gpmnb^+^Spp1^+^ and IFNRM subpopulations recede after the emergence of massive regenerating myofibers at day 7 to day 14 [44,78,81] (Figure 2). These findings strongly suggest that the sequential appearance of specialized macrophage subpopulations may be critical for the proper muscle injury repair. In addition, none of these macrophage subpopulations conform to strict M1 or M2 activation status [81]. Both Ly6C^hi^ and Ly6C^lo^ populations are heterogeneous: monocytes, pro-inflammatory, and IFNRM subpopulations are Ly6C^hi^, which are more pro-inflammatory in general, while the other subpopulations are Ly6C^lo^, which are more anti-inflammatory in general [81].

### 4.2. Both Resident and Infiltrating Macrophages Play Pro-Regenerative Roles in Regulating Acute Skeletal Muscle Injury Repair

Following acute skeletal muscle injury, resident macrophage populations are quickly overwhelmed by the massively infiltrated blood monocytes-derived macrophage populations, and they account for only a minor fraction of intramuscular macrophages [81]. Despite the minor presence, a recent study indicated that resident macrophages play an indispensable role in acute skeletal muscle injury repair [36]. Depleting TIM4^+^Lyve1^+^ resident macrophages early in the acute injury repair process leads to an extended persistence of necrotic fibers and hinders muscle regeneration. Consequently, this self-renewing resident macrophage subtype is necessary for the efficient removal of damaged tissue to facilitate proper regeneration [36]. Indeed, single cell-based transcriptomic analysis revealed that TIM4^+^Lyve1^+^ resident macrophages upregulate genes related to phagocytosis and inflammatory cell recruitment post-injury, indicating a potential additional role in triggering the inflammatory response [36]. However, the depletion of this specific subset did not alter the influx of either neutrophils or monocytes [36], suggesting possible compensation by other resident cells.

Infiltrating macrophages spatiotemporally regulate key processes for complete repair after acute muscle injury, including inflammation, myogenesis, and ECM remodeling. Impairing the recruitment or timely phenotypic transition of infiltrating macrophages hinders muscle regeneration, which is often accompanied by deposition of fibrofatty tissue [10,35,43,44,45,46,66,67]. These observations imply that the resident macrophage population is unable to compensate for the roles of infiltrating macrophages to support complete repair of acutely injured skeletal muscle.

Infiltrating macrophages regulate both the propagation and the resolution of inflammatory response during acute muscle injury repair. scRNAseq analysis shows the presence of a pro-inflammatory macrophage subpopulation at an early stage, producing a high level of pro-inflammatory mediators, and inflammation-resolution macrophage subpopulations at a later stage, producing a high level of inflammation-resolution mediators after acute muscle injury [78,81]. In addition to producing inflammatory mediators, the early infiltrated Ly6C^hi^ macrophages are also required to clear damaged muscle debris, as blocking macrophage infiltration by CCR2 deficiency delays the clearance of necrotic muscle fibers [44]. Phagocytosis of damaged tissue debris has been shown to drive the pro- to anti-inflammatory phenotype switch of macrophages [35,49,50,51,52,53].

Infiltrating macrophages regulate myofiber regeneration by orchestrating the activation, proliferation, differentiation, and fusion of myogenic cells post-injury. These functions appear to be distributed among distinct macrophage subtypes. Pro-inflammatory macrophages primarily enhance the activation and proliferation of myogenic cells, whereas anti-inflammatory macrophages support their differentiation and fusion [35,82,83,84]. An early study showed that conditioned media from pro-inflammatory activated, in vitro cultured macrophages greatly enhanced the proliferative potential of human primary myoblasts, indicating a role of soluble factors secreted by pro-inflammatory macrophages [85]. Subsequent studies further identified that pro-inflammatory macrophage-derived factors, including fibronectin [86], IL-6 [87], TNF-α [88], PGE2 [89], and A Disintegrin-Like and Metalloproteinase with Thrombospondin Type 1 Motif (ADAMTS1) [90], can stimulate MuSC activation and proliferation. In contrast, anti-inflammatory macrophages likely promote myoblast differentiation and myofiber growth through factors like IL-4 [91], IGF-1 [44,92,93], and GDF-3 [94]. Notably, the Gpnmb^+^Spp1^+^ macrophage subpopulation identified by scRNAseq analysis highly expresses pro-regenerative factors GDF-15 and GPNMB [79,95] and resides with regenerating myofibers [95], suggesting an important role of this macrophage subpopulation in myofiber regeneration. Additionally, the shift in macrophage phenotype during repair is associated with increased glutamine synthesis, which further enhances satellite cell activation and regeneration [96]. Therefore, the temporal dynamics of different macrophage phenotypes appear critical for proper muscle regeneration. This concept is supported by studies showing that disruption of key signaling molecules involved in macrophage phenotypic switching, including IGF-1 [93], Meteorin-like (Metrnl) [97], AMP-activated protein kinase-1 (AMPKα1) [49,98], Nuclear Factor IX (Nfix) [52], CCAAT/enhancer binding protein-β (C/EBPβ) [72], and peroxisome proliferator-activated receptor-γ (PPARγ) [94], impairs the regenerative process.

Infiltrating macrophages are key regulators of ECM remodeling during acute skeletal muscle injury repair. ECM remodeling is a tightly controlled process of temporary ECM deposition and degradation that provides crucial structural support for activating and differentiating myogenic cells. Beyond this scaffolding function, specific ECM components, including collagen 6a (Col6a) [99] and fibronectin [86], can directly activate muscle stem cells (MuSCs). The main producers of ECM are fibro/adipogenic progenitors (FAPs), which also aid myogenic cell activation and differentiation to promote regeneration [5,100,101,102]. However, when the repair process is disrupted, abnormal FAP activation can occur, leading to fibrofatty tissue accumulation and failure to support MuSC activation [102]. Macrophages modulate both the number and activity of FAPs in injured muscle. Pro-inflammatory macrophages secrete TNF-α to induce FAP apoptosis, thereby restricting excessive accumulation [103]. Conversely, anti-inflammatory macrophages can promote the proliferation of fibrogenic cells by producing pro-fibrotic factors such as TGF-β1 [104]. The scRNAseq-identified Gpnmb^+^Spp1^+^ macrophage subpopulation around day 3 post-acute injury features signature genes of scar-associated macrophages (SAMs) [81]. SAMs are found in multiple tissues and organs with physical proximity to excessive ECM, expressing genes regulating ECM deposition [105,106,107]. Therefore, Gpnmb^+^Spp1^+^ macrophages, aside from their potential role in myofiber regeneration, may also play an important role in ECM remodeling during acute skeletal muscle injury repair. Furthermore, depleting macrophages or inhibiting their recruitment not only hinders muscle regeneration but also promotes fibrosis [43,103], underscoring the essential role of infiltrating macrophages in controlling FAP behavior and ECM remodeling.

In summary, both resident macrophages and adequately infiltrated blood monocytes-derived infiltrating macrophages are critically required for proper acute skeletal muscle injury repair. Driven by the change of microenvironment in the injured muscle, infiltrating macrophages undergo temporal, heterogeneous activation and differentiate into functionally diverse subpopulations, which differentially regulate inflammation, myofiber regeneration, and ECM remodeling in a spatiotemporal manner.

## 5. Heterogeneous Macrophage Activation in Muscular Dystrophy

### 5.1. Duchenne Muscular Dystrophy (DMD)

Muscular dystrophies are a diverse group of genetic diseases characterized by progressive muscle atrophy and weakness [108], among which the most studied is Duchenne muscular dystrophy (DMD). DMD is caused by the defective dystrophin gene on the X chromosome [109], which disrupts the dystrophin–glycoprotein complex, leading to a fragile muscle cell membrane and subsequent muscle fiber deaths [110]. This genetic defect triggers a repeated cycle of muscle degeneration and regeneration, causing chronic inflammation and the replacement of muscle tissue with fibrous and fatty deposits [111]. DMD patients die prematurely from respiratory and cardiac muscle weakness [108,109]. Research on DMD primarily uses animal models, most commonly *mdx* mice. *Mdx* mice exhibit a less severe phenotype than human DMD patients [112,113,114,115]. In these mice, muscle inflammation begins around 3 weeks of age, continues until 2–3 months, and then gradually subsides in limb muscles but persists in the diaphragm [55,112,113,114,115]. Accordingly, progressive fibrosis develops in the diaphragm but not in the limb muscles. The progressive diaphragm fibrosis impairs respiratory muscle function in *mdx* mice, resembling the condition in human patients [55,114,116,117]. We will focus our following discussion on studies with *mdx* mice, as comprehensive study with DMD patient muscles is lacking.

### 5.2. Co-Existence of Heterogeneous Monocyte/Macrophage Subpopulations in mdx Muscles

Studies targeting macrophages in *mdx* muscles have generated controversial results. Depletion of macrophages at 4 weeks of age reduces limb muscle necrosis [118], likely due to reduced inflammation-induced muscle damage. Similarly, reduction of macrophage infiltration by germ-line deficiency of CCR2 in *mdx* mice decreases muscle damage and fibrosis while it improves muscle function in both limb muscle and diaphragm before 3 months of age [56,57]. Infiltrating Ly6C^hi^ macrophages are also shown to contribute to the fibrosis of *mdx* leg muscles at 8 to 10 weeks of age by producing latent TGF-β1 [119]. On the other hand, an independent study depleting macrophages from *mdx* limb muscles at 10 to 12 weeks of age impairs myogenic cell proliferation and differentiation, decreases myofiber formation, and increases fibrofatty tissue deposition [120]. This controversy may be due to the different ages when macrophage depletion is performed, as the activation status of intramuscular macrophages in *mdx* muscles may vary in different ages.

Recent studies with single cell-based transcriptomic analysis and spatial transcriptomic analysis have revealed heterogeneous activation of macrophages underlying their pleotropic roles in *mdx* muscles [58,81,95,121,122]. Despite batch differences, these studies identified similar macrophage subpopulations: 1) pro-inflammatory monocyte/macrophage subpopulation; 2) pro-fibrotic/pro-regenerative Gpmnb^+^Spp1^+^ macrophage subpopulation; 3) IFN-activated macrophages; 4) macrophage populations resembling muscle resident macrophages; and 5) proliferating macrophages [58,81,95,121,122]. Data obtained from *mdx* limb muscles, including both early age (4 weeks) and older age (14 weeks), show that the pro-inflammatory monocytes/macrophages and Gpmnb^+^Spp1^+^ macrophages are the majority of intramuscular macrophages at 4 weeks [122], while resident-like macrophages are the majority at 14 weeks [81]. Comparative analysis among wild-type uninjured, acutely injured, and *mdx* muscles indicates that the pro-inflammatory monocytes/macrophages and Gpmnb^+^Spp1^+^ macrophages are injury-associated subpopulations that are present in injured, but not uninjured, muscles [81]. The predominance of these two monocyte/macrophage subpopulations in 4-week-old *mdx* limb muscle is therefore indicative of a high level of muscle damage. As blocking Ly6C^hi^ monocytes infiltration by CCR2 deficiency reduces muscle damage [56,57], the highly pro-inflammatory monocytes/macrophages may also contribute to muscle damage at early age. Meanwhile, the pro-fibrotic/pro-regenerative Gpmnb^+^Spp1^+^ macrophages may be actively repairing the damaged myofibers. This speculation is supported by the study with spatial transcriptomics analysis, which shows multilayered regenerative inflammation zones where the pro-inflammatory monocytes/macrophages reside with necrotic myofibers while the Gpmnb^+^Spp1^+^ macrophages reside with regenerating myofibers [95]. In 14-week-old *mdx* mice, monocytes/macrophages contain similar subpopulations in the diaphragm compared to limb muscle, with a significantly higher fraction of pro-inflammatory monocytes/macrophages in the diaphragm than in limb muscle [58,81]. This finding suggests a higher degree of damage in the diaphragm than in limb muscle, possibly contributing to the persistent inflammation in *mdx* diaphragm.

### 5.3. The Persistence of Pro-Inflammatory Monocytes/Macrophages and Gpnmb^+^Spp1^+^ Macrophages May Contribute to Chronic Inflammation and Progressive Fibrosis in mdx Muscles

The direct comparison of monocytes/macrophages between acutely injured and *mdx* muscles did not identify distinct pathogenic monocyte/macrophage subpopulations in dystrophic muscle [81]. Instead, the injury-associated pro-inflammatory monocytes/macrophages and Gpnmb^+^Spp1^+^ macrophage subpopulations persist in dystrophic muscle but not in acutely injured muscle [78,81,95,122]. The persistence of these two subclusters, which reflects continuous monocyte infiltration and differentiation that is triggered by constant muscle injury in dystrophic muscle, may contribute to chronic inflammation and progressive fibrosis (Figure 2). The pro-inflammatory monocytes/macrophages may contribute to myofiber damage, which further induces inflammation. The Gpnmb^+^Spp1^+^ macrophages may also play an important role in fibrosis in addition to their potential roles in myofiber regeneration. This macrophage subpopulation features the expression of signature genes of SAMs, including *Gpnmb*, *Spp1*, *Fabp5*, *Trem2*, *Cd9*, and *Cd63*, which are reportedly associated with fibrogenesis across different tissues [105,106,107,123,124,125,126,127]. The *Spp1* gene encodes osteopontin, which has been increasingly recognized for its involvement in the progression of tissue fibrosis [128]. Global genetic deletion of osteopontin in *mdx* mice leads to reduced intramuscular TGF-β, reduced fibrosis in the diaphragm, and increased muscle regeneration and strength [129]. Macrophage-specific deletion of *Spp1* in *mdx* leads to reduction in an adipogenic stromal cell population, reduced intramuscular fat accumulation, and improved muscle function [130]. These in vivo findings support the notion that the Gpnmb^+^Spp1^+^ macrophages may contribute to the progressive fibrofatty tissue replacement in dystrophic muscle via, at least in part, the osteopontin expression. In line with this hypothesis, spatial transcriptome analysis has shown that the Gpnmb^+^Spp1^+^ macrophages are associated with stromal cells and ECM genes in the dystrophic muscle of *mdx* mice [122]. In addition to *Spp1*, macrophage-specific deletion of *Trem2*, another featured gene of Gpnmb^+^Spp1^+^ macrophages, has been reported to reduce fibrosis in mouse models of pulmonary fibrosis [131] and myocardial infarction [132]. These together add additional support to the potential fibrogenic roles of Gpnmb^+^Spp1^+^ macrophages in *mdx* muscles.

## 6. Suppressing Intramuscular Monocyte/Macrophage Accumulation as a Potential Therapy for DMD

Preclinical studies have demonstrated that ameliorating inflammation and fibrosis improves DMD muscle function [133,134,135,136,137,138,139]. Currently, the main therapeutic approach mitigating chronic inflammation and fibrosis in DMD is the use of glucocorticoids [140,141,142] (Table 1). The efficacy of these treatments has been shown to be limited, and chronic use of corticosteroids can lead to significant side effects, including reduced growth, weight gain, and osteoporosis [143,144]. Developing new pharmacotherapy to target inflammatory cells is needed. Monocytes/macrophages are the predominant inflammatory cells in *mdx* muscles [55]. The persistence of the injury-associated pro-inflammatory monocytes/macrophages and Gpnmb^+^Spp1^+^ macrophages in *mdx* muscles likely contribute to the pathological progression [78,81,95,122]. Therefore, targeting monocytes/macrophages may represent a viable approach to treat DMD.

Accumulation of monocytes/macrophages in *mdx* muscles mostly results from blood monocytes infiltration [58], which relies on CCR2 signaling [56,57]. Therefore, targeting CCR2 signaling may be viable to reduce the accumulation of monocytes/macrophages to alleviate the pathology of *mdx* muscles. Interestingly, CCR2 deficiency in *mdx* mice diminishes both Ly6C^hi^ and Ly6C^lo^ monocytes/macrophages in dystrophic muscles before 3 months of age, while it only diminishes Ly6C^hi^ monocytes/macrophages, but not Ly6C^lo^ macrophages, after 3 months. Correspondingly, the diaphragm pathology and function are improved at 3 months but not 6 months [56,57]. Thus, blocking Ly6C^hi^ monocyte/macrophage infiltration via CCR2 deficiency does not provide a sustained improvement of *mdx^5cv^* diaphragm dystrophy. The beneficial effects are lost after the expansion of intramuscular Ly6C^lo^ macrophages. A recently published study suggests that these expanded Ly6C^lo^ macrophages are pathogenic [58]. They express a similar level of genes promoting inflammation and fibrosis, such as *Ccl2*, *Tgfb1*, *Spp1*, and *Mmp14*, compared to the infiltrating macrophages in age-matched *mdx^5cv^* muscles [58]. Moreover, the fraction of the pro-fibrotic Gpnmb^+^Spp1^+^ macrophages [105,122,123,124] in *mdx^5cv^/Ccr2^−/−^* muscles is similar to that in age-matched *mdx^5cv^* muscles [58]. Therefore, it appears necessary to suppress both Ly6C^hi^ monocyte/macrophage infiltration and Ly6C^lo^ macrophage expansion to achieve sustained reduction in macrophages and improvement in muscle dystrophy. To achieve this, the source of the Ly6C^lo^ macrophage expansion in *mdx^5cv^/Ccr2^−/−^*muscles must be elucidated. In the same published study, the authors further provide evidence that the proliferation of pre-existing skeletal muscle resident macrophages, particularly those of embryonic origin, contributes to the Ly6C^lo^ macrophage expansion when Ly6C^hi^ monocyte/macrophage infiltration is blocked in *mdx^5cv^/Ccr2^−/−^* muscles [58]. Moreover, the proliferation of muscle resident macrophages in *mdx^5cv^/Ccr2^−/−^* muscles may be driven by an increased intramuscular level of colony-stimulating factor 1 (CSF1), which is mainly expressed by FAPs [58]. Consistently, it has been shown in an independent study that the FAP-derived CSF1 is required for the survival of self-renewing skeletal muscle resident macrophages [146]. Long-term inhibition of the CSF1 receptor (CSF1R) with pharmacological inhibitor effectively depletes muscle resident macrophages in *mdx* muscles [36]. However, no notable effect on myofiber size or diaphragm maximum tetanus force production is observed [36]. This is likely due to the compensation by blood monocytes-derived infiltrating macrophages, as it has been reported that CSF1R inhibition does not significantly impact blood monocytes [147] or monocyte-derived infiltrating macrophages [36,148]. Therefore, dual inhibition of CCR2 and CSF1R to suppress both the infiltration of inflammatory monocytes/macrophages and the pathogenic expansion of resident macrophages may be needed to persistently reduce the accumulation of intramuscular monocytes/macrophages to potentially provide sustained improvement of the pathology and function of DMD muscles.

## 7. Conclusions

In both acutely injured and dystrophic muscles, accumulation of monocytes/macrophages is a prominent feature of inflammation. Comprising both blood monocytes-derived infiltrating macrophages and pre-existing resident macrophages, intramuscular macrophages are heterogeneously activated in response to the microenvironment changes in injured muscles, regulating inflammation, myofiber regeneration, and ECM deposition. During the regenerative repair of acutely injured muscle, the heterogeneously activated monocyte/macrophage subpopulations are present in temporal dynamics, coordinating with the muscle injury repair process. In particular, the injury-associated monocyte/macrophage subpopulations recede along with the emergence of the inflammation resolution macrophage subpopulations. In contrast, the injury-associated monocyte/macrophage subpopulations persist in dystrophic DMD muscles where the muscle injury is chronic and secondary to genetic defects. This difference is likely the cause of the chronic inflammation and progressive fibrosis seen in dystrophic muscles but not in acutely injured muscles. Resident macrophages in the DMD mouse model undergo expansion and pathogenic activation when inflammatory monocyte infiltration is inhibited. While limiting myofiber damage by gene, cell, and pharmacological therapies represents a main treatment strategy, controlling persistent macrophage accumulation by dual suppression of inflammatory monocyte infiltration and resident macrophage expansion may provide a useful adjunct therapy for DMD. This hypothesis needs further testing.

## Figures and Tables

**Table 1:** Anti-inflammatory treatments in clinical use for DMD.

Name	Mechanism	FDA approval	Application
Prednisone [140]	Synthetic glucocorticoid	Off-label use	Standard treatment
Deflazacort [140]	Oxazoline derivative of prednisolone	Approved in 2017	Patients aged 5 years and older
Vamorolone [140,142]	Anti-inflammatory steroid analogue	Approved in 2023	Patients aged 2 years and older
Givinostat [145]	Histone deacetylase (HDAC) inhibitor	Approved in 2024	Patients aged 6 years and older

## Data Availability

No new data were created or analyzed in this study. Data sharing is not applicable to this article.

## Abbreviations

The following abbreviations are used in this manuscript:

ADAMTS1	A Disintegrin-Like and Metalloproteinase with Thrombospondin Type 1 Motif
AMPKα1	AMP-activated protein kinase-1
CCL2	CC motif chemokine ligand 2
CCR2	CC motif chemokine receptor 2
C/EBPβ	CCAAT/enhancer binding protein-β
Col6a	Collagen 6a
CSF1	Colony-stimulating factor 1
CSF1R	Colony-stimulating factor 1 receptor
CX3CL1	C-X3-C motif chemokine ligand 1
CX3CR1	C-X3-C motif chemokine receptor 1
DMD	Duchenne muscular dystrophy
ECM	Extracellular matrix
FACS	Flow cytometry analysis
FAPs	Fibro/adipogenic progenitors
GDF-3	Growth differentiation factor-3
GDF-15	Growth differentiation factor-15
GPNMB	Glycoprotein nonmetastatic melanoma protein B
HSCs	Hematopoietic stem cells
IFNRM	Interferon-responsive macrophages
IGF-1	Insulin-like growth factor-1
IL-4	Interleukin-4
IL-6	Interleukin-6
ISGs	IFN-responsive genes
Ly6C	Lymphocyte antigen 6 complex locus C
Metrnl	Meteorin-like
MHCII	Major histocompatibility complex class II
MuSCs	Muscle satellite cells
Nfix	Nuclear Factor IX
PGE2	Prostaglandin E2
PPARγ	Peroxisome proliferator-activated receptor-gamma
SAMs	Scar-associated macrophages
scRNAseq	Single cell-based RNA sequencing
TGF-β1	Transforming growth factor-beta 1
TIM4	T-cell immunoglobulin and mucin domain containing 4
TLF	*Timd4* and/or *Lyve1* and/or *Folr2*
TNF-α	Tumor necrosis factor-alpha
